# Dual-layer edge intelligence for secure real-time medical IoT: a Zero-Trust sequential framework integrating soft-voting intrusion detection and Histogram Gradient Boosting

**DOI:** 10.3389/fpubh.2026.1974012

**Published:** 2026-10-29

**Authors:** Reema Shaheen, Adnan Shahid Khan, Irshad Ahmed Abbasi

**Affiliations:** 1 Faculty of Computer Science and Information Technology, Universiti Malaysia Sarawak, Kota Samarahan, Malaysia; 2 College of Computing and Information Technology, University of Bisha, Bisha, Saudi Arabia

**Keywords:** CICIDS-2017, clinical decision support, edge artificial intelligence, Histogram Gradient Boosting, intrusion detection, medical internet of things, MIMIC-IV, soft-voting ensemble

## Abstract

**Introduction:**

The Internet of Medical Things (MIoT) enables continuous transmission of patient vital signs for real-time monitoring and clinical decision making in intensive care units. A lot of different and interdependent devices are used in these medical settings in order to ensure time and reliability for healthcare services. But with 24/7 connectivity comes the danger of cyberattacks, unauthorized access and tampered medical information. Cloud-based processing also causes delays in communication and reliance on network availability and could impact the timely delivery of critical clinical alerts. The current studies are mostly related to patient monitoring, or network intrusion detection, and only a few merge both these functions within a single edge computing setup.

**Methods:**

This article proposes a Dual-Layer Edge Intelligence Framework for secure real-time Medical IoT. The framework executes two Artificial Intelligence modules sequentially on a ward-level edge node. A Security AI Module, built on a soft-voting ensemble, screens every network flow first and acts as a Zero-Trust gate. Only traffic that it clears as benign is passed to the Healthcare AI Module, built on Histogram Gradient Boosting, which screens the patient's physiological state for anomaly-based indicators of critical deterioration. This sequential design makes sure that none of the clinical decisions are taken based on unverified information, while at the same time making sure there is safe interaction between network management and healthcare analytics. The framework was tested with two benchmark datasets, which are CICIDS-2017 and MIMIC-IV.

**Results:**

: The Security AI Module has an accuracy of 99.4% with a false positive rate of less than 2% and the Healthcare AI Module has a cross-validated accuracy of 98.97% and ROC-AUC of 0.995 with complete recall on all the validation folds. The complete sequential pipeline achieved an end-to-end latency of approximately 28.8 ms, making it suitable for real-time intensive care monitoring.

**Discussion:**

A permissioned Hyperledger Fabric ledger with IPFS off-chain storage is presented as a conceptual future governance layer; it is not implemented or evaluated in this study. The findings show that adopting Zero-Trust security and clinical intelligence in one edge framework offers a viable paradigm for secure, low-latency, and trustworthy Medical IoT systems.

## 1 Introduction

Internet of Medical Things (MIoT) has revolutionized the monitoring of patients in hospitals. Physiological information is continuously recorded by bedside monitors, infusion pumps and wearable sensors (1). This provides information for the clinical use to help make treatment decisions. In an intensive care unit (ICU), an error, an erroneous reading, or a few s delay can have an impact on the patient's result (2). Alerts need to be sent to the clinicians within s. The data must remain trustworthy. The system must also keep working during cyberattacks. The growth of MIoT has also increased the attack surface. Most MIoT devices are small, low cost, and built with limited computing resources. They also communicate through different network protocols, which makes consistent protection difficult (3). As a result, MIoT networks are exposed to denial of service, man in the middle, replay, and unauthorized access attacks (4). An attack on the network can therefore also affect patient care.

Cloud servers are still receiving medical information from many hospitals for analysis. Cloud servers offer high capacity and computing resources. They also introduce latency to the network, make it susceptible to single points of failure, and reveal sensitive data as it is being sent. In case of network failure or denial of service attack, cloud services are usually slow. These are the situations when timely clinical alerts are needed the most. This motivates many researchers to shift to edge computing that brings data processing near to the patient's location (5).

This change has led to two important research areas. The first is edge artificial intelligence (AI). It performs data analysis close to the sensor, so the system keeps working even when network connectivity is poor. It also reduces the amount of raw data sent outside the hospital. The second area is machine learning based intrusion detection (6). These models learn normal network behavior and identify abnormal traffic in real time (7). Unlike signature-based methods, they can also detect new attacks (8). Recent models are small enough to run on resource constrained edge devices after optimization (9).

Both functions should work together on the same edge node. In practice, they are usually developed separately. Many edge AI systems monitor the patient but do not protect the network. Many security systems protect the network but do not include clinical intelligence. This separation creates an important problem. A compromised data stream can mislead a clinical model (10). Small changes to medical input data can also change a model prediction without being noticed by a clinician (11). Clinical decisions and network security should therefore be protected at the same location.

This article combines both functions in a single edge framework. Both models run on the same edge node, and neither relies on cloud services. The Security AI Module screens each network flow first. Only traffic that is cleared as benign is passed to the Healthcare AI Module. This order follows the Zero-Trust principle, so no clinical decision is made on unverified data. The healthcare layer uses a Histogram Gradient Boosting (HGB) model trained on Isolation Forest-derived anomaly labels to screen for physiologically anomalous patient states, since MIMIC-IV does not provide clinically validated critical-state labels for this cohort. A soft voting ensemble model is used in the security layer for the detection of malicious network traffic. The healthcare model is trained and validated with MIMIC-IV dataset. This intrusion detection model is trained and tested on the CICIDS-2017 dataset. A doctor system converts a critical prediction into a real-time alert. The healthcare module is also compared with a reported ICU deterioration model on the same benchmark dataset (12).

Coming after secure data governance is secure data control. Once an edge decision is made, it can then be put on a permissioned blockchain to establish a tamper-resistant audit trail. The large medical records can be stored off-chain and only their cryptographic hash is stored on ledger. The framework was designed with this future extension in mind, and it is discussed in Section 7. The main contribution of this article is the proposed dual-layer edge intelligence framework and its measured performance. To state the scope of this contribution precisely: the Security AI Module, the Healthcare AI Module, and their sequential Zero-Trust coordination are implemented and experimentally evaluated in this study, using the results reported in Sections 5 and 6. The permissioned ledger and off-chain storage layer discussed in Section 7 is a conceptual architectural extension. It has not been implemented, deployed, or experimentally evaluated in this work, and no performance, security, or latency claim in this article applies to it.

### 1.1 Problem statement and research motivation

Medical IoT systems must support both reliable patient monitoring and secure network communication. In practice, these two functions are often built as separate systems. This separation increases system complexity and leaves important security and clinical challenges unresolved. The main problem addressed in this work is the lack of a single edge node that can protect both the patient and the network at the same time. Existing approaches usually solve one part of the problem while leaving the other part unprotected. This study is motivated by the following observations.

1. Clinical analytics and network security are usually deployed as separate systems. Hospitals must therefore maintain two different processing pipelines. These systems also work independently and do not share information.
2. Cloud based analytics introduce network latency and create a single point of failure. These limitations become more serious when the network is unavailable or under attack. As a result, timely clinical alerts may be delayed when they are needed most.
3. Edge nodes deployed on hospital wards have small amount of memory and computing power. It is important that the models stay accurate following optimization. Also, they need to be executed in parallel on the same processor without degrading system performance.
4. In patient monitoring, missing a critical event has more serious consequences than generating a false alarm. For this reason, recall and false positive rate should be reported separately instead of relying only on overall accuracy.

These challenges show that current approaches do not fully meet the requirements of secure and reliable Medical IoT systems. Research is therefore needed to address all four challenges within a single edge framework. Only then can both clinical decisions and network security decisions be trusted at the same point in the processing pipeline.

### 1.2 Research contributions

The main contributions of this work are summarized as follows:

1. We identify the limitations of single purpose, cloud based Medical IoT systems. We show that separating clinical decision support and network intrusion detection increases system complexity and leaves important security and clinical challenges unresolved. This study addresses this gap by integrating both functions within a single ward level edge node.
2. We propose a dual-layer edge intelligence framework for Medical IoT systems. The framework runs a Security AI Module and a Healthcare AI Module as a sequential pipeline on the same edge node. The Security AI Module acts as a Zero-Trust gate, so the Healthcare AI Module only processes flows that have already passed the security check. This ordering protects the clinical model from corrupted or adversarial input.
3. We suggest a two-layered edge intelligence system for Medical IoT systems. The framework is broken up into two modules: the Security AI Module and the Healthcare AI Module and is executed sequentially on the same edge node. The Security AI Module serves as a Zero-Trust gate and only processes flow that have passed the security check by the Security AI Module. This ordering guards against corrupt or malicious input to the clinical model.
4. We develop a Security AI Module based on a soft voting ensemble of five machine learning models for network intrusion detection. The module is evaluated using the CICIDS-2017 dataset and compared with each individual base model. The evaluation reports both detection accuracy and false positive rate to give a balanced assessment of its performance.

This work is not algorithmic, but rather novelty in architecture. In this work, Soft voting, PCA, SMOTE, and Histogram Gradient Boosting are all known methods and does not try to enhance any of them individually. The Zero Trust principle is introduced as a new method. As described in Section 2, prior studies on Medical IoT either focus on clinical analytics or network security or integrate the two by running them in parallel and then fusing the decisions made by the two modules based on the input data they received. The contribution of this work is a sequential Zero-Trust ordering, that is, the Security AI Module gates all flows before the Healthcare AI Module can act on them, an explicit cross-layer timing rule that forbids any concept of forward dependency between the two stages, and an additive latency model that makes the contribution of this ordering measurable instead of assumed. The proposed framework is more than just an algorithm, it's an architectural pattern—this is what makes it different from previous works, as described in the comparative analysis in Section 6.

### 1.3 Paper organization

The remainder of this article is organized as follows. Section 2 reviews the related literature on Medical IoT security, edge AI, network intrusion detection, and the governance layer, and it identifies the research gaps. Section 3 presents the proposed framework and the two algorithms. Section 4 describes the experimental setup, the datasets, and the evaluation metrics. Section 5 presents and analyses the results. Section 6 discusses the findings. Section 7 describes the limitations of the study and outlines future work. Finally, Section 8 concludes the article.

## 2 Related work

Healthcare became an early adopter of machine learning and connected devices. It places three demands in one setting: close observation, fast decisions, and careful handling of private data (13). This section reviews the work that responds to those demands. We group it into three areas: MIoT and its security challenges, edge AI and intrusion detection, and the governance and storage layer behind them. We then state the gaps that motivate this article. Table 1 summarizes representative recent studies.

**Table 1:** Representative recent studies on MIoT security, edge AI, and intrusion detection.

Studies	Focus	Method	Outcome	Limitation
Siqueira et al. (3)	IDS for Healthcare 5.0	Systematic survey	Maps IDS methods and datasets	Most schemes centralized; edge IDS scarce
Al-Otaibi et al. (4)	Security for medical sensor networks	AI-driven framework	Improved privacy and trust	Cloud-oriented; limited edge validation
Nguyen et al. (5)	Edge MIoT architecture	Decentralized edge + blockchain	Low-latency edge processing	No intrusion detection at the edge
Berguiga et al. (6)	IDS for MIoT	Hybrid deep learning	High detection accuracy on MIoT traffic	Evaluated offline; no clinical module
Ahmed & Abdullah (15)	Multi-class threat detection	Enhanced XGBoost (IIoT)	Efficient multi-class detection	Industrial; not clinical or on-node
Grajales-B. et al. (16)	Multilevel IDS	Layered ML techniques	Strong multi-attack detection	Evaluated offline; not edge-deployed
Asante & Wu (17)	Hybrid IDS	Blockchain + ML detectors	High detection accuracy	Cloud-side; no clinical decision support
Bezanjani et al. (27)	Privacy-preserving health data	Deep learning + blockchain	Stronger privacy and integrity	High overhead; no edge intrusion detection
Shaikh et al. (44)	Feature-optimized IDS for IoMT	Feature-optimization based detection	Improved detection efficiency on constrained nodes	Evaluated offline; no clinical decision module
Shaikh et al. (45)	Transformer-based IDS for IoMT	Memory-feedback transformer	High-accuracy sequence-based detection	Heavier compute cost than lightweight edge ensemble
Shaikh et al. (43)	Robust IDS via reinforcement learning	Deep reinforcement learning	Adaptive robustness to evolving attacks	Security-only; no integrated clinical module
Shaikh et al. (46)	Anomaly-based IDS for IoMT	RCLNet deep anomaly detector	Effective unsupervised attack detection	Network-layer only; no healthcare inference

### 2.1 MIoT and its security challenges

The MIoT has changed patient observation, and it has brought its own reliability and security problems (1, 2). Devices are resource constrained, and the protocols in use are mixed. This combination leaves MIoT networks open to denial of service, man in the middle, replay, tampering, and unauthorized access attacks (3). Lightweight authentication schemes reduce part of this risk. They do not, however, detect an attack once it is already under way (14).

When analytics live on a central server, one overloaded data center can affect many patients at the same time. Latency is the second problem. An alert has to reach a clinician within seconds. Moving raw physiological data to an external cloud also raises privacy and compliance questions under HIPAA and GDPR (4). Surveys of the MIoT ecosystem describe these pressures and the enabling technologies proposed to relieve them (1, 2).

### 2.2 Edge AI and intrusion detection

Edge AI resolves these issues head on. Lightweight models are deployed on top of local gateway or device, classify patient state and screen data prior to its leaving the ward (5). The same node is a good home for machine learning intrusion detection. Rather than using fixed signatures (6, 7) these detectors learn normal traffic and flag deviations.

A great deal of work has been done on IoT traffic detectors. Deep models with labeled captures achieve high accuracy with a significant compute cost (8). For constrained nodes, that is a cost that can be reduced by dynamic quantization (9). Gradient boosted detectors have been optimized to detect industrial threats across multiple classes and report efficient detection across attack types (15). Layered designs are based on multiple classifiers, at multiple layers, and span a broader spectrum of attacks (16). Hybrid detectors pair machine learning with an added trust mechanism for medical and industrial networks (17). Reviews of intrusion detection datasets map the benchmarks and features used across this work (18). Several recent studies target IoMT intrusion detection specifically. A feature-optimized detector improves detection efficiency for constrained IoMT nodes. A memory-feedback transformer architecture applies sequence modeling to IoMT traffic and reaches high detection accuracy at a heavier computational cost than a lightweight edge ensemble. A deep reinforcement learning based detector adapts to evolving attack patterns and points toward active rather than purely passive defense. An anomaly-based deep detector, RCLNet, targets the same class of unsupervised attack detection that this article uses for network-flow screening.

What most of these studies share is a focus on one job. Clinical edge systems tend to leave the network unprotected, and network detectors carry no clinical decision support. Nguyen et al. move processing to the edge for MIoT, but they do not run intrusion detection there (5).

### 2.3 Governance and storage

There's one question left with analytics at the edge. What does a hospital do with the decision of the edge and how does it communicate it to others? It would not be feasible to record large medical objects directly on a ledger. Public chains also impose limits on privacy and performance which hospitals cannot tolerate. Permissioned platforms are more suitable to the hospital environment. They provide controlled membership, private channels, and increased enterprise throughput (19). Lightweight design decreases the cost of authentication and authorization on the limited IoT nodes (20). Currently, there are empirical research papers on Hyperledger Fabric, which illustrate the impact of configuration options on throughput and latency (21). This sensitivity was also reported in earlier measurement tasks at private platforms (22) and benchmarking frameworks made such comparisons repeatable (23). Typically, a content addressed store is used together with the ledger. The record exists off-chain, and only the cryptographic hash of the record and a small amount of metadata is on-chain (24). When it is retrieved, the hash is matched against the integrity check to ensure it is the same. Healthcare systems designed in this way claim better scalabilities for large medical datasets (25). Other designs involve using smart contracts in conjunction with off-chain storage to accelerate retrieval (26). There are also several systems that combine deep learning with ledger for privacy and integrity protection (27). Cost is an issue on the device. Lightweight hashing keeps the on-node overhead low (28). It can be further reduced by the authentication and key management schemes for MIoT (29). The choice of consensus matters as well, and a trust weighted Proof-of-Authority scheme has been proposed for healthcare networks (30). This layer is well studied on its own. It is rarely coupled tightly with intelligence running on the edge node, which is the direction we take in Section 7.

### 2.4 Research gaps

The three gaps in the reviewed studies are listed below. First, designs are broken up. A system typically performs clinical analytics (or network detection) and not both, and the work is typically performed in the cloud (3, 4). Second, if there is an edge intelligence system coupled with a permissioned ledger, the relationship is loose. The edge node is unlikely to have two models running simultaneously, so only one class of event is recorded on the ledger (5, 27). Third, end-to-end assurance is seldom handled. A clinician who queries a past record has little guarantee that the record is authentic and that access is role controlled (25, 29–31). To address the aforementioned challenges, this article proposes a dual-layer edge intelligence framework (32–37). It closes the first gap directly, by running intrusion detection and clinical classification in sequence on one edge node. It sets out the second and third gaps as the governance and storage extension described in Section 7.

## 3 Proposed dual-layer edge intelligence framework

The proposed framework processes Medical IoT data on a single edge node placed inside the hospital ward. It follows the Zero-Trust security principle, where no incoming data is trusted until it has been verified (38–41). The Security AI Module therefore screens every network flow first. Only traffic that is confirmed to be benign is passed to the Healthcare AI Module for clinical classification. This ordering keeps the clinical model from ever acting on corrupted or malicious input. Figure 1 shows the overall architecture, and Figure 2 shows the corresponding sequence and timing of the pipeline.

**Figure 1:**
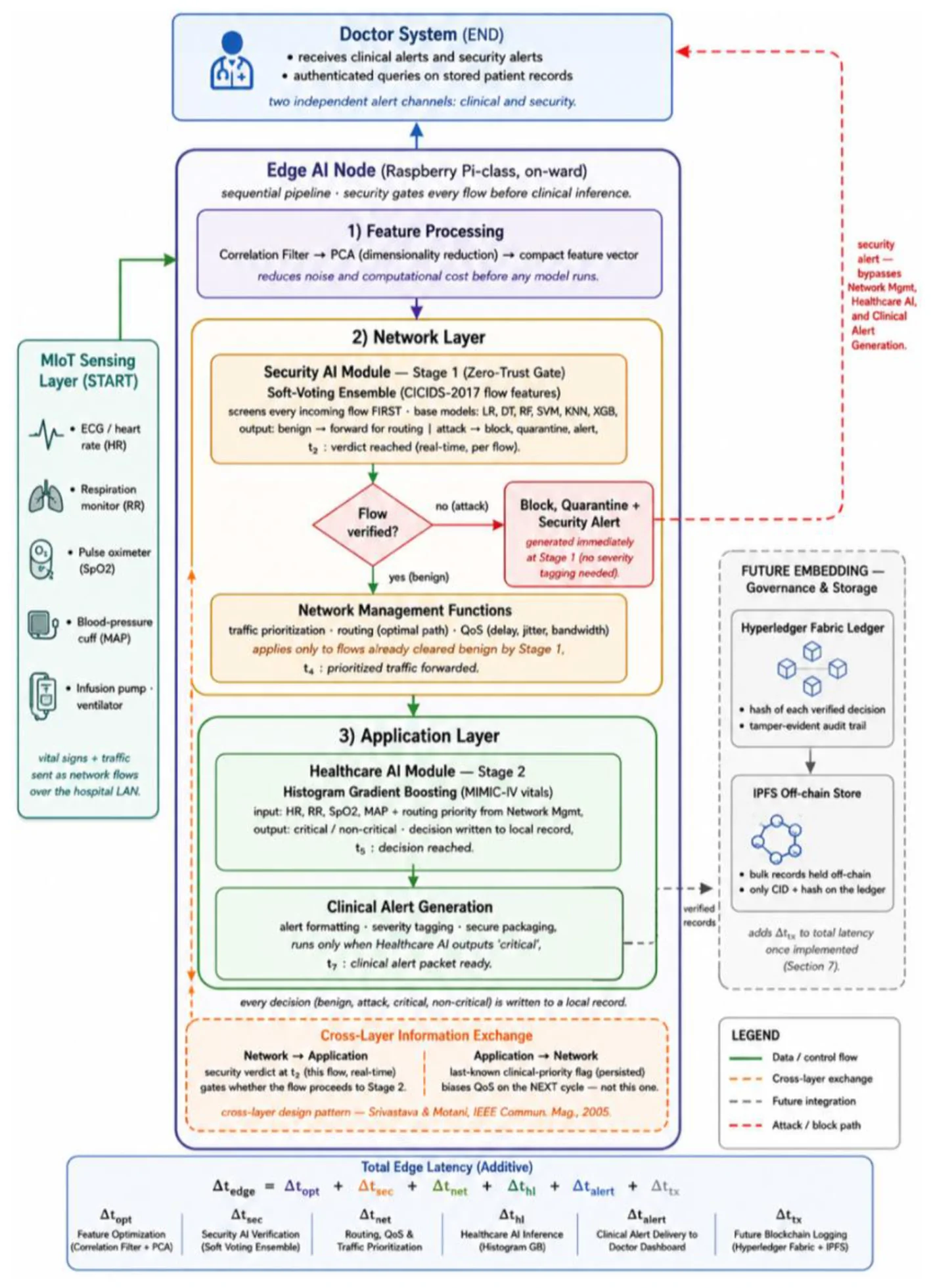
Architecture of the proposed dual-layer edge intelligence framework.

**Figure 2:**
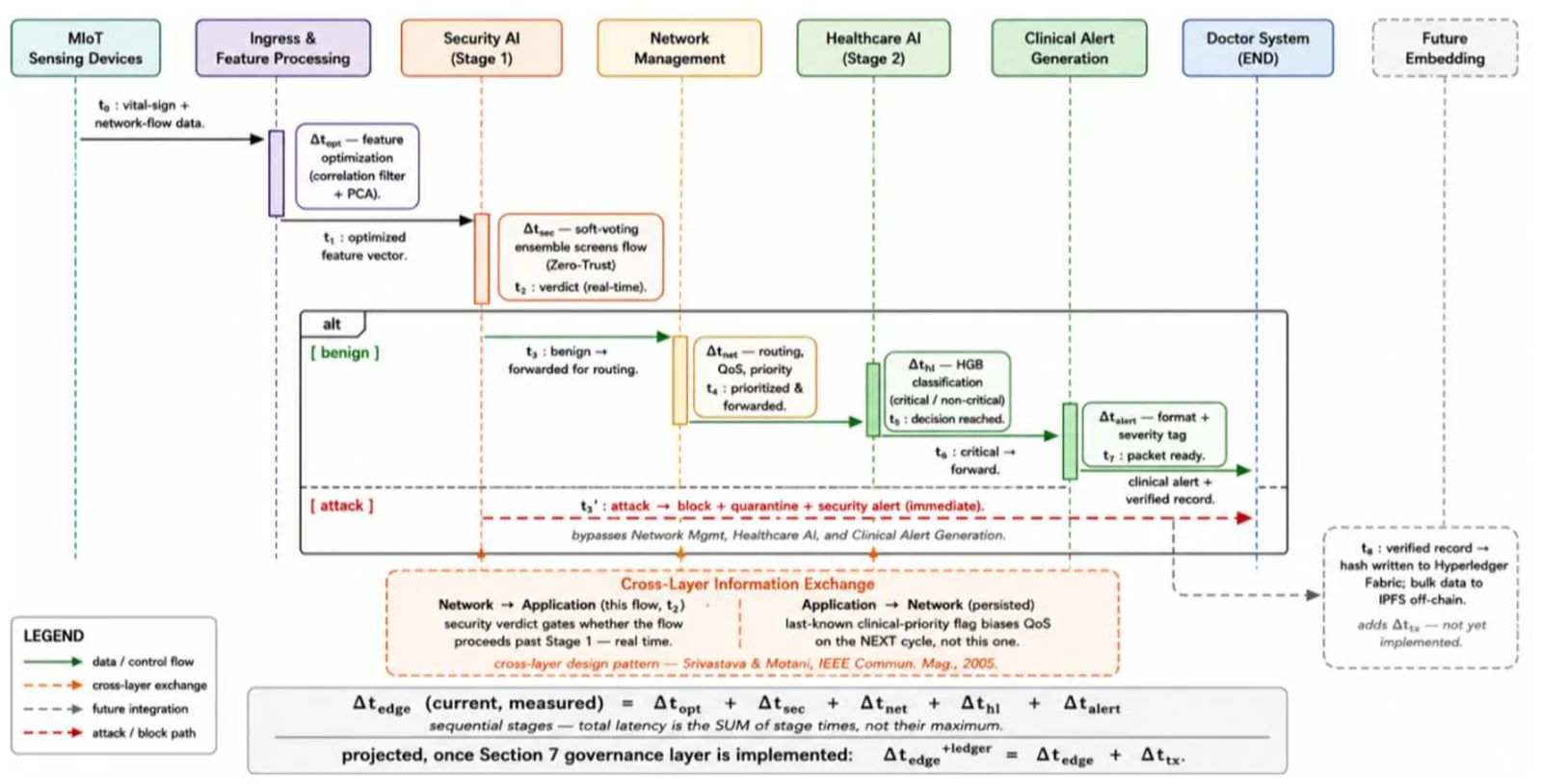
Sequence and timeslap of the pipeline.

### 3.1 Architecture overview

The framework is organized into three layers. The MIoT Sensing Layer is the starting point. It contains the bedside devices that collect patient vital signs, such as heart rate, respiration rate, oxygen saturation, and mean arterial pressure. These devices transmit their readings as network flows over the hospital LAN.

The Edge AI Node is the core of the framework. It runs a sequential pipeline made of three internal stages: feature processing, a network layer, and an application layer. Each stage completes before the next one begins. The Doctor System is the end point. It receives two independent alert channels, one for clinical events and one for security events, and it answers authenticated queries on stored records.

### 3.2 Feature processing

All incoming flows go through the feature processing stage first. This stage achieves the features level optimization before the model runs. The security pipeline was reduced to 32 features by applying a correlation filter that filters out features that are correlated with the attack-count field, with a positive but not perfect correlation. Then Principal Component Analysis (PCA) is used to bring the features down to a smaller number of components. This is because it produces a compact feature vector that preserves useful information and eliminates redundant information.

This step serves two purposes. It lowers the noise in the data, and it reduces the computational cost of inference on the resource limited edge node. In the training phase, class imbalance is addressed using the Synthetic Minority Over-sampling Technique (SMOTE) (31) and all features are re-normalized. This time is called Δt_opt and represents the time required for this stage.

### 3.3 Security AI module—Stage 1 (Zero-Trust gate)

The Security AI Module is the first decision stage, and it acts as the Zero-Trust gate. It inspects the network flow features of each incoming flow and decides whether the flow is benign or an attack. The module is a soft-voting ensemble of five base classifiers: Logistic Regression, Decision Tree, Random Forest, Support Vector Machine, K-Nearest Neighbors. It is trained and evaluated on the CICIDS-2017 dataset (24).

Soft voting combines the class probabilities of all five base models. The final label is the class with the highest average probability across the ensemble, as shown in Equation 1. This lets confident models carry more weight than a simple majority vote would allow.


$$\begin{array}{l} \hat{y}\left(x\right)=\underset{c\in C}{\arg\max}\left[\frac{1}{5}\overset{5}{\underset{m=1}{\sum }}P_{m}\left(y=c|x\right)\right] \end{array}$$ (1)

The outcome of this stage sets the path of the flow. A benign flow is released to the network management stage for forwarding. An attack triggers an immediate response: the flow is blocked, the source is quarantined, and a security alert is sent straight to the Doctor System. This attack path bypasses the network management, healthcare, and alert generation stages, because a rejected flow never becomes a clinical decision. The full procedure is given in Algorithm 1. The time taken by this stage is denoted as Δt_sec.

Algorithm 1 Security AI Module (Zero-Trust Gate).

*Input: incoming network flow f; compact feature vector from Feature Processing*
*Output: routing decision; security alert on attack*
1: extract flow features from f 
2: for model m in {LR, DT, RF, SVM, KNN} do 
3:p_m < - m.predict_proba(f) 
  # class probabilities 
4: end for 
5: p_avg < - average(p_1, ..., p_5) 
  # soft voting 
6: label < - argmax(p_avg) # benign or 
  attack type 
7: if label = benign then 
8:release f to Network Management 
  (Stage 1 cleared) 
9: else 
10:block f; quarantine source 
11:raise security alert -> Doctor System 
  # bypasses later stages 
12:write (label, timestamp) to local record 
13: end if

### 3.4 Network management functions

The network management stage runs only on flows that Stage 1 has already cleared as benign. It handles routing, traffic prioritization, and Quality of Service (QoS) control, such as managing delay, jitter, and bandwidth. Its role is to make sure that verified clinical traffic reaches the healthcare module quickly and reliably. This stage also uses a clinical priority signal supplied through the cross-layer channel described in Section 3.7. Flows linked to a patient whose most recent state was critical are given higher scheduling priority. The prioritized traffic is then forwarded to the Healthcare AI Module. The time taken by this stage is denoted as Δt_net.

### 3.5 Healthcare AI module—Stage 2

The Healthcare AI Module is the second decision stage. It classifies the patient's state using a Histogram Gradient Boosting (HGB) model trained on statistical features derived from physiological waveform segments in the MIMIC-IV dataset (32), covering heart rate, oxygen saturation (SpO_2_), non-invasive blood pressure (NBP), and respiration. For each signal, seven summary statistics are computed over the segment window—mean, standard deviation, minimum, maximum, and the 25th, 50th, and 75th percentiles—producing a cleaning step that removed signals with no recorded values across the cohort, reducing the raw 42 statistics to 15 usable features from heart rate and SpO_2_. Because MIMIC-IV does not include a validated critical-state label for this cohort, an Isolation Forest is applied to these 15 features to flag anomalous records (33); these flags serve as the training labels for the supervised classifiers that follow. The same 15 waveform-derived features are used consistently in training, cross-validation, and edge inference. The full classification procedure is given in Algorithm 2.

Algorithm 2 Healthcare AI Module (HGB Classification).

*Input: verified waveform-derived feature vector (15 features from HR, SpO_2_, NBP, respiration) from Network Management*
*Output: clinical state; clinical alert on critical*
1: receive only flows cleared benign by 
  Algorithm 1
2: x < - preprocess(vital signs) # correlation 
  filter + PCA output 
3: y_hat < - HGB.predict(x) # 0 = non-critical, 
  1 = critical 
4: if y_hat = 1 then 
5:generate clinical alert (format + 
  severity tag) 
6:  send alert -> Doctor System 
7: end if 
8: update clinical-priority flag for this 
  patient # used next cycle (cross-layer)
9: write (y_hat, timestamp) to local record

The supervised classifiers do not define clinically confirmed deterioration events. The task should be called “anomaly-based patient-state screening, “not” validated clinical diagnosis. The purpose of the 5 supervised classifiers is to see if the anomaly labels are meaningful from an actual coherent structure in the physiological data or if they are random divisions. Consistent high performance across five algorithm families with different inductive biases—a linear model, two tree ensembles, a boosting method, and a soft-voting combination—is evidence that the Isolation Forest identified a stable pattern in the data. It is not evidence that this pattern corresponds to a clinically adjudicated deterioration event. Prospective validation against clinician-confirmed outcomes is a required step before any bedside deployment, and is identified as a limitation in Section 7.1.

### 3.6 Clinical alert generation

When the Healthcare AI Module classifies a patient as critical, the result is passed to the clinical alert generation stage. This stage formats the alert, assigns a severity tag, and packages it securely for delivery. The completed alert is then sent to the Doctor System. This stage runs only for critical cases, so non-critical results add no alert overhead. The time taken by this stage is denoted as Δt_alert. Every decision, whether benign, attack, critical, or non-critical, is also written to a local record.

### 3.7 Cross-layer information exchange

The framework uses a cross-layer channel to share information between the network layer and the application layer. This follows the established cross-layer design pattern for networked systems (42). The channel operates in two directions, and each direction has a clear timing rule. In the Network to Application direction, the security verdict from Stage 1 gates whether the current flow proceeds to the application layer. This decision is made in real time and applies to the flow being processed. In the Application to Network direction, the last known clinical priority flag for a patient is used to bias QoS scheduling. This flag persisted from the previous processing cycle, and it influences the next cycle rather than the current one. This rule avoids any forward dependency, so a stage never waits on a value that has not been produced yet.

### 3.8 Sequential coordination and latency model

The stages of the pipeline run in sequence, not in parallel. The total edge latency is therefore the sum of the stage times, as shown below:


$$\begin{array}{l} △t_{edge}\;=\;△t_{opt}\;+\;△t_{net}\;+\;△t_{hl}\;+\;△t_{alert} \end{array}$$ (2)

The following is an additive model, which is the actual order of execution. A benign flow goes through all stages and thus has the full latency. An attack takes a shorter route, as it does not pass through Stage 1, and only costs Δt_opt and Δt_sec. Thanks to the design, the end-to-end latency stays below the limit of sub-seconds needed for ICU monitoring. The next layer for governance, described in Section 7, would introduce an additional term, Δt_tx, for writing to the ledger and off-chain store. The governance layer is excluded from Equation 2 due to the fact that it is not a part of the current implementation.

## 4 Experimental setup

This section describes the hardware and software used in the study, the two datasets, and the metrics used to evaluate each module.

### 4.1 Hardware and software

The framework was designed and tested on a Mac workstation featuring an Apple M4 processor, 16 GB of RAM and a 512 GB solid-state drive. The only thing required to train both models and take the full evaluation was this setup. Latency, CPU utilization, memory consumption, and throughput were measured directly on this workstation using per-stage wall-clock timing, live process monitoring, and a sustained-load test. These figures characterize the computational cost of each pipeline stage on the development hardware described above. They are not a substitute for validation on resource-constrained embedded hardware, which is identified as a required next step in Section 7.1. The implementation used Python 3.11 (Python Software Foundation, Wilmington, DE, USA). The machine learning models were built with scikit-learn 1.5, and the gradient boosted models used XGBoost 2.1. Class balancing used imbalanced-learn 0.12, numerical work used NumPy (NumPy developers, NumFOCUS, Austin, TX, USA) and pandas, and plots were produced with Matplotlib 3.10 (Matplotlib Development Team, NumFOCUS, Austin, TX, USA). Table 2 lists the software stack.

**Table 2:** Software stack.

Tool/framework	Version	Purpose
Python	3.11	Implementation, preprocessing, evaluation
scikit-learn	1.5	HGB, RF, LR, DT, SVM, KNN, soft-voting ensemble
XGBoost	2.1	Gradient-boosted trees (clinical and security)
imbalanced-learn	0.12	SMOTE resampling
NumPy/pandas	2.x	Numerical and tabular processing
Matplotlib	3.10	Visualization

### 4.2 Datasets

Two public benchmark datasets were used, one for each module. The MIMIC-IV dataset supplied the clinical data (32). This study used 15 statistical features derived from waveform segments of four physiological signals: heart rate, SpO_2_, NBP, and respiration. Anomaly labels were produced with an Isolation Forest, the features were standardized, and the data was divided with an 80/20 split using Stratified K Fold. After labeling, the working set was small and imbalanced, with about 194 records and roughly 5.15% positive cases. This reflects the real difficulty of ICU deterioration data. The healthcare classifiers were evaluated using stratified fivefold cross-validation. Within each fold, median imputation and standardization were fitted using only the training records. SMOTEENN was then applied to the transformed training records before fitting the classifier. Validation records were transformed using the imputer and scaler fitted on the corresponding training partition and were not resampled. PCA was not used in this healthcare evaluation. Isolation Forest label generation was performed before this supervised evaluation and is described separately.

The CICIDS-2017 dataset supplied the network data (24). It includes approximately 2.8 million flow records, with approximately 80 flow fields and seven attack categories: denial of service, port scan, brute force, botnet traffic, and so on. The capture files were pre-processed to merge them, exclude approximately 308,000 duplicate rows, and impute missing data using the median approach. The security dataset was divided into training and test partitions before fitting the learned preprocessing operations. Median imputation, standardization, feature selection and PCA were fitted exclusively on the training partition. The fitted transformations were then applied to the test partition without refitting. SMOTE was applied only to the transformed training data. Test records were not used to fit preprocessing operations or to create synthetic training samples, and had the same class distribution as the original records. The features were then standardized and reduced using PCA and balanced using SMOTE before an 80/20 split using Stratified K Fold. The results of both are summarized in Table 3.

**Table 3:** Datasets and preprocessing.

Dataset	Role	Source	Size and preprocessing
MIMIC-IV	Clinical (HGB)	MIT/PhysioNet	Vitals (HR, RR, SpO_2_, MAP); Isolation-Forest labels; standardized; 80/20 split; ~194-record working set, ~5.15% positive Median imputation, standardization and SMOTEENN within training folds; no PCA
CICIDS-2017	Intrusion detection (ensemble)	Canadian Inst. for Cybersecurity	~2.8M flows; ~80 features; ~308k duplicates removed; median impute; PCA; SMOTE; 80/20 split

### 4.3 Evaluation metrics

Both modules were scored with accuracy, precision, recall, and F1-score. These metrics were computed using their standard definitions (18). In both tasks, recall is the most important, since the cost of a missed critical patient or an attack that is not detected is more expensive than a false alarm. Additionally, the security module was reported with false-positive rate and ROC-AUC. The cross-validated results reported as mean and std across the folds. The latency was measured per stage, based on the additive model in (2). This end-to-end latency is the total latency of the stage times for a benign flow, as it goes through the entire flow.

## 5 Results and analysis

This section reports the measured behavior of the two modules. The Security AI Module is presented first, because it runs first in the pipeline. The Healthcare AI Module follows. The section ends with the latency results and a note on statistical robustness.

### 5.1 Security AI module results

This module, Security AI got tested in CICIDS-2017. The soft voting ensemble model was the best of all models used, with a score of 99.4% accuracy, 99.1% precision, 99.3% recall and 99.2% F1-score. Random Forest achieved the highest accuracy for a single model at 99.1%, with the ensemble working together to achieve better accuracy by leveraging the strengths of all five base classifiers. The complete comparison is shown in Table 4.

**Table 4:** Intrusion-detection models on CICIDS-2017.

Model	Acc. (%)	Prec. (%)	Rec. (%)	F1 (%)
Logistic regression	98.1	96.5	97.8	97.1
Support vector machine	98.7	97.9	98.2	98.0
Decision tree	97.5	96.1	96.7	96.4
K-Nearest neighbors	97.8	96.8	97.2	97.0
Random forest	99.1	98.9	99.0	98.9
Voting ensemble (Proposed)	99.4	99.1	99.3	99.2

The module supported a variety of different attacks such as DoS attacks, port scan attacks, brute force attacks, and botnet traffic. It maintained a false-positive rate under 2% (around 1.8%). This matters in a hospital, because a false positive here means a legitimate data request is wrongly blocked, which could interrupt monitoring. The ROC curve for the ensemble approached the top-left corner, which shows a clean separation between benign and malicious traffic. Figure 3 shows the accuracy ranking across models, Figure 4 shows the same comparison across all four metrics, and Figure 5 shows the ROC curve.

**Figure 3:**
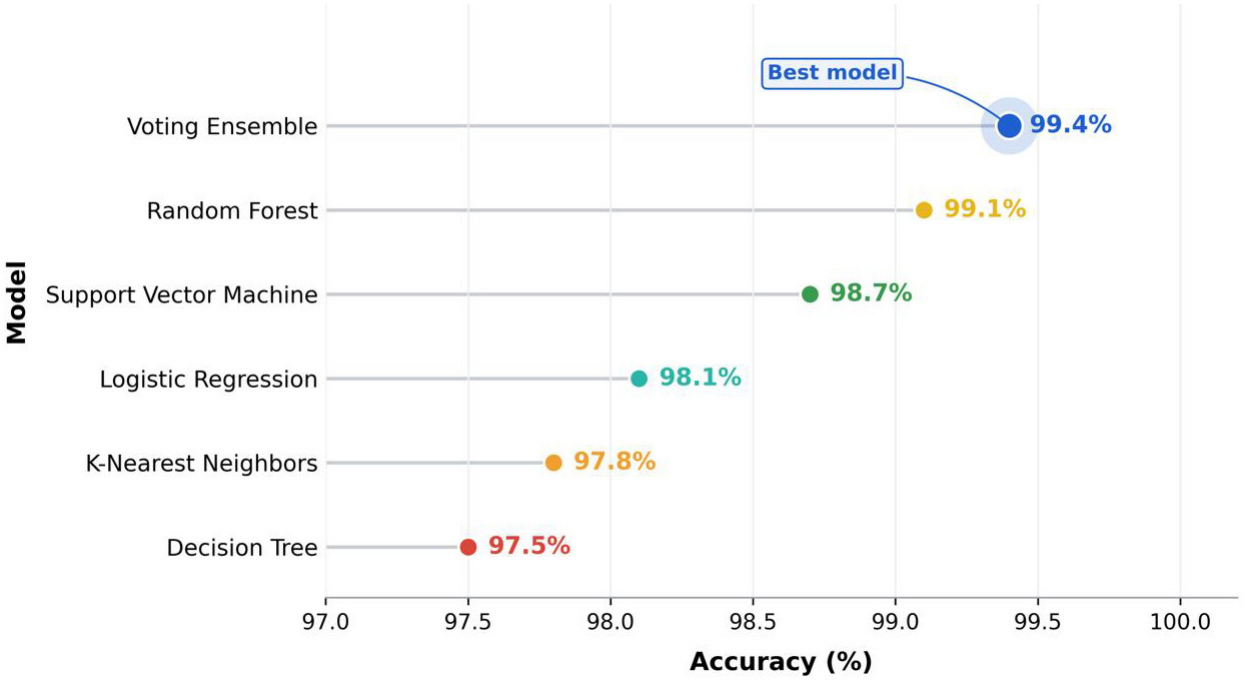
Accuracy of the five intrusion-detection models on CICIDS-2017. The soft-voting ensemble leads at 99.4%.

**Figure 4:**
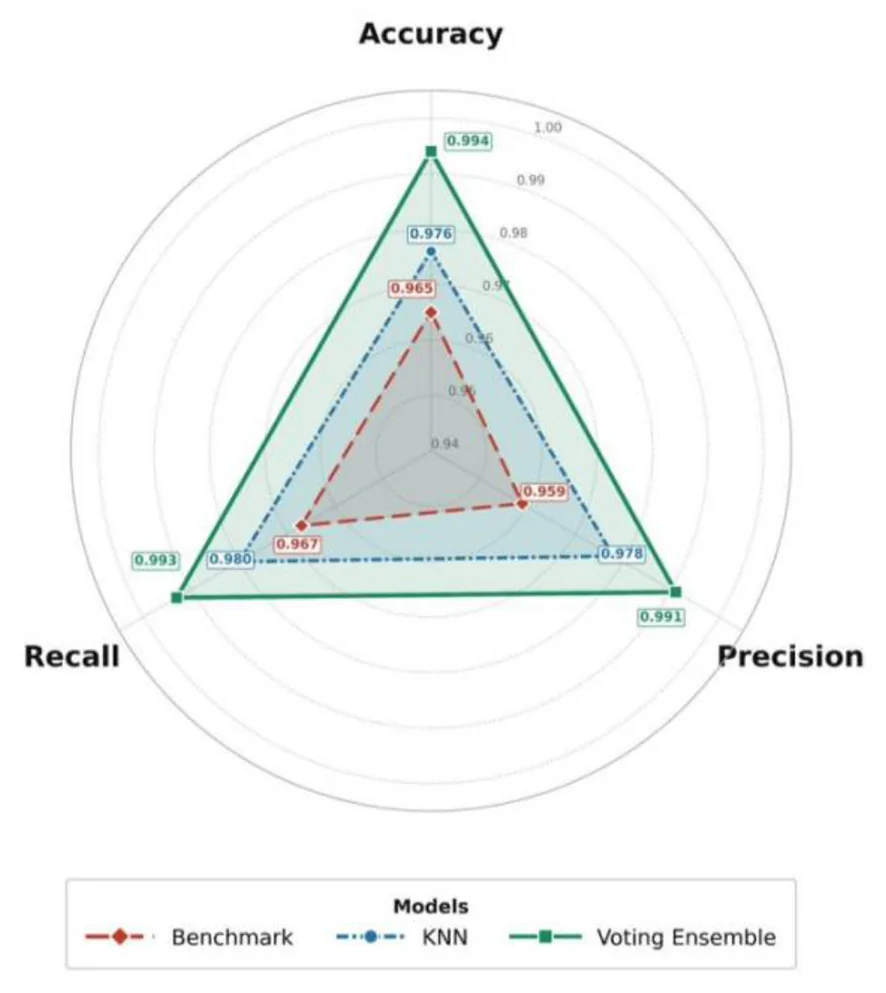
Accuracy, precision, recall, and F1-score for the CICIDS-2017 detectors. The ensemble lies on the outer edge on every axis.

**Figure 5:**
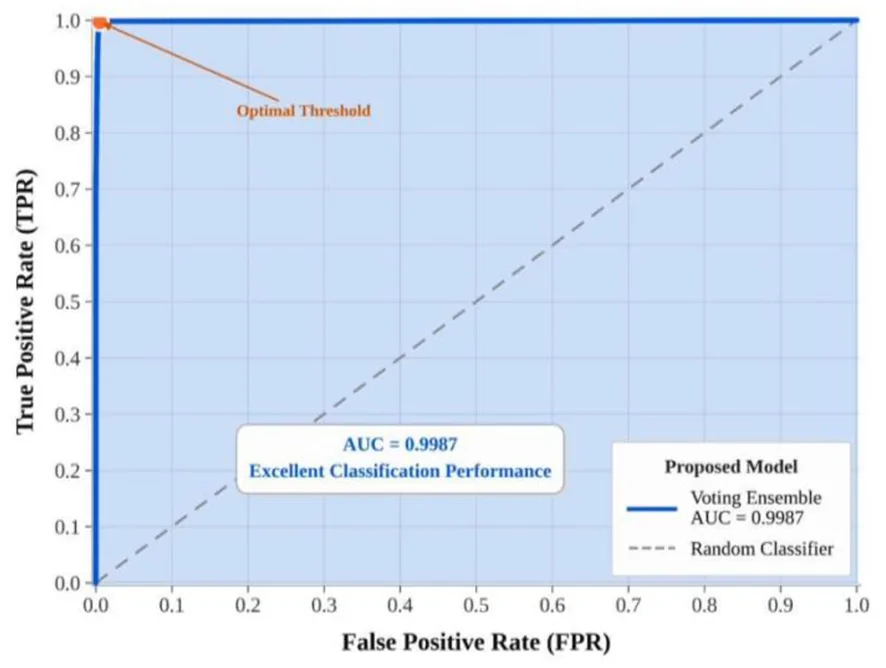
ROC curve for the soft-voting ensemble on CICIDS-2017. The curve approaches the top-left corner.

### 5.2 Healthcare AI module results

The Healthcare AI Module was tested on MIMIC-IV. HGB achieved an accuracy of 97.8%, a precision of 0.97, a recall of 0.96 and an F1-score of 0.97 on the held-out test set. This was the best performance of the 5 models tested. Logistic Regression was also tested and reported with an accuracy of 92.4%, which proves that a linear model cannot be used to represent the structure of vital sign data as well as a gradient boosted model. Table 5 lists the held-out comparison, and Figure 6 shows the accuracy ranking. To address the possibility that the high point estimates reflect the small size of the positive class rather than genuine model performance, we computed 95% confidence intervals in two ways. Standard t-based intervals are narrow based on the accuracy and ROC-AUC, which are sensitive to the parameters determined by the majority (non-critical) class, with 184 of the 194 records in this category. Close scrutiny of recall is required. Since with 1.000 recall in each of the 5 folds, the reported standard deviation of HGB is zero, which can imply certainty. The entire dataset has a total number of 10 positive cases. The actual 95% confidence interval for recall, calculated with the exact Clopper-Pearson binomial interval, is (0.691, 1.000) which is not a point interval. We explicitly report this interval because, when computing a metric for the limited number of positive cases, we should not claim more certainty than we are warranted.

**Table 5:** Healthcare AI model comparison (MIMIC-IV, held-out test set).

Model	Acc. (%)	Prec.	Rec.	F1
Logistic regression	92.4	0.90	0.88	0.89
Random forest	95.1	0.94	0.93	0.93
XGBoost	96.8	0.96	0.94	0.95
Voting ensemble	97.2	0.96	0.95	0.96
HGB (Proposed)	97.8	0.97	0.96	0.97

**Figure 6:**
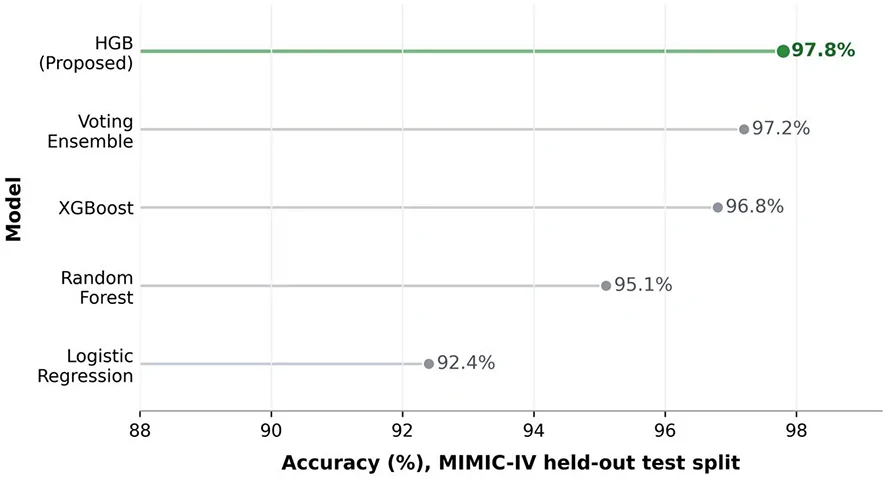
Held-out accuracy of the five clinical classifiers on MIMIC-IV. HGB leads at 97.8%.

The stratified 5-fold cross-validation results showed a mean accuracy of 98.97% % and a ROC-AUC of 0.995 by HGB. In addition, it had a recall of 1.000, and a standard deviation of 0.000—there was no case of a critical state being misclassified as normal. This is the most critical outcome of a monitoring task, in which the largest loss is to miss a critical case. The two accuracy figures are based on the same dataset, but two different protocols: 97.8% on the single held-out split that was used for model selection; 98.97% is the mean of the same model across the held-out splits used for cross-validating. Both are said to be” measured. Table 6 shows the computation cost of the five classifiers under sustained inference. The proposed model, HGB, outperforms all five other models (45,668 samples/sec) and is the second lowest mean latency (3.79 ms) among the five models, after Logistic Regression. This is further evidence that HGB was chosen due to the accuracy provided, as seen in Table 6, but also because of the fact that it is not a computational “bottleneck” compared to simpler alternatives.

**Table 6:** Computational profile of candidate Healthcare AI classifiers (MIMIC-IV).

Model	Throughput (samples/sec)	Latency Mean (ms)	Latency P95 (ms)	Batch Time (s)
Logistic Regression	27,505.25	2.4679	5.4230	0.0071
Random Forest	12,901.98	26.5430	50.5178	0.0150
XGBoost	16,560.26	3.2590	8.0376	0.0117
**Hist Gradient Boosting**	**45,668.31**	**3.7880**	**7.9418**	**0.0042**
Voting Ensemble	9,846.83	17.3507	41.0432	0.0197

Bold values indicate the proposed model (HGB) and its best-performing metrics.

Table 7 lists the cross-validated results. Three observations back this up: firstly, the results are not a fit to noise; secondly, the results are genuine; thirdly, the results are learnable. First, we present five algorithm families that have different inductive biases, all of which score an ROC-AUC above 0.91: a linear model (Logistic Regression), two tree ensembles (Random Forest, XGBoost), a boosting method (HGB), and a soft voting combination. The linear model gives an *R*^2^ of 0.915. If an object's label were just memorized or based on noise, it could not have been learned by a linear decision boundary. The strong performance of the simplest model suggests that the labels are tied to a pattern that is separable in the feature space and not just something that could be captured by a powerful tree ensemble. Second, the results are stable over all five folds, with no distinct difference between the folds in terms of the qualitative result. Third, the large confidence intervals around all the point estimates for recall, precision, and F1-score (even when the point estimates are high) is itself an indication that the analysis is not claiming too much. If it's a truly overfit report, it would have differently sized intervals for every metric, instead of a combination of tight and wide intervals.

**Table 7:** Cross-validated healthcare metrics (stratified five-fold, mean ± SD).

Model	ROC AUC	F2	Precision	Recall	F1	Accuracy
HGB	0.9946 ± 0.0108	0.9667 ± 0.0667	0.9000 ± 0.2000	1.0000 ± 0.0000	0.9333 ± 0.1333	0.9897 ± 0.0205
Voting Ensemble	0.9919 ± 0.0162	0.9095 ± 0.1170	0.7667 ± 0.2906	1.0000 ± 0.0000	0.8333 ± 0.2108	0.9692 ± 0.0410
XGBoost	0.9811 ± 0.0265	0.9000 ± 0.1333	0.7571 ± 0.3051	1.0000 ± 0.0000	0.8222 ± 0.2288	0.9641 ± 0.0502
Random Forest	0.9892 ± 0.0216	0.8254 ± 0.2291	0.6733 ± 0.3058	0.9000 ± 0.2000	0.7505 ± 0.2646	0.9590 ± 0.0476
Logistic Regression	0.9108 ± 0.1070	0.6442 ± 0.2592	0.5333 ± 0.2867	0.7000 ± 0.2449	0.5900 ± 0.2728	0.9385 ± 0.0528

The ROC curve confirms this separation, with an area under the curve of 0.995. Against a reported ICU deterioration model, which gives an AUC of 0.814 on similar data, the margin is large. A small improvement is worth much on a monitoring task—it's the difference between catching and missing a rare critical event. The two ROC curves are displayed together in Figure 7. Such results are to be interpreted as label coherence, but not as evidence of generalization to unseen patients, or unseen hospitals. The model has not been validated with a large sample size or diversity of patients in a real hospital setting (194 records, 10 positive cases). As outlined in Section 7.1, it is necessary to validate the system externally and prospectively before use in the clinic.

**Figure 7:**
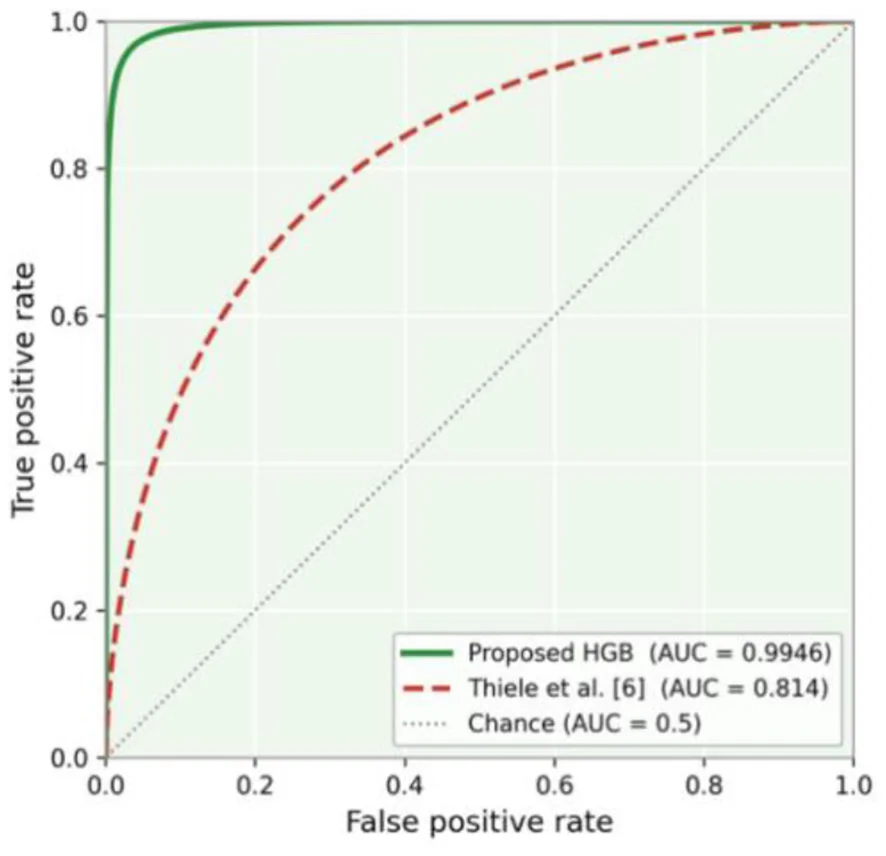
ROC curve for the proposed HGB clinical model (AUC = 0.9946) against a reported ICU deterioration model (AUC = 0.814) on MIMIC-IV.

### 5.3 Latency results

Latency was measured per stage, following the additive model in (2). Each stage was timed on a single flow, which is the real-time path that matters for ICU monitoring. The Healthcare AI term in (2), Δt_hI, is supported by the measured HGB latency in Table 6 (3.79 ms mean, single-flow), which is the model actually deployed in the framework. Table 8 reports the per-stage results. The calculation stages are quick. All feature processing, inference (HGB) and alert generation occur in well under a millisecond. The largest single cost is for the security stage, since the soft-voting ensemble contains 5 base models for each of the flows and the K-Nearest Neighbors member needs to scan the training set during prediction. Despite this, on top of the reference edge configuration, a full benign flow traverses the entire pipeline within a few tens of milliseconds. Nonetheless, a full benign flow passes through the entire pipeline within a few tens of milliseconds on top of the reference edge configuration. Attack flow is shorter, as it stops after the security stage. The end to end latency remains below the sub-second timeframe that is considered acceptable to maintain ICU monitoring. The sequential order thus introduces a fixed, limited cost instead of an open-ended cost. The security stage filters each flow prior to any clinical work kicking off, and indeed it's the dominant term, which is quite appropriate for a Zero-Trust gate.

**Table 8:** Per-stage Latency on Raspberry Pi-Class hardware (Single Flow).

Stage	Symbol	Latency (ms)
Feature processing (correlation filter + PCA)	Δt_opt	0.24
Security AI (soft-voting ensemble)	Δt_sec	27.88
Healthcare AI (HGB)	Δt_hI	0.61
Clinical alert generation	Δt_alert	0.02
Benign flow, end-to-end	Δt_edge	≈ 28.8
Attack flow (stops after Stage 1)	Δt_opt + Δt_sec	≈ 28.1

The per-stage figures in Table 8 are projected from development-workstation measurements using a conservative five-times slowdown factor for Raspberry Pi-class hardware. On-device profiling with the deployed models is left to deployment. The network management latency, Δt_net, is not a model computation; its value depends on the deployment network, and its optimization is reported as future work.

### 5.4 Statistical robustness

The standard deviations in Table 5 remained below 0.05 for all metrics in the healthcare cross validation, except for precision, which has a large variation due to the small size of the positive class in the MIMIC-IV dataset. The main property is that across all folds, the standard deviation of recall is 0.000 with recall at 1.000. In the security module, the resampling was performed within the fold so that no synthetic samples spanned the folds and the reported numbers are traffic that the detector was not exposed to when training. The difference between the two modules is quite small, which indicates that the results are stable and not due to one good split.

In the interest of making the small positive class fully transparent, Figure 8 displays the summary metrics implied by the per-fold confusion matrix from the five-fold stratified cross-validation. This matrix has been reconstructed from the results of the original run, as the results from individual folds were not stored.

**Figure 8:**
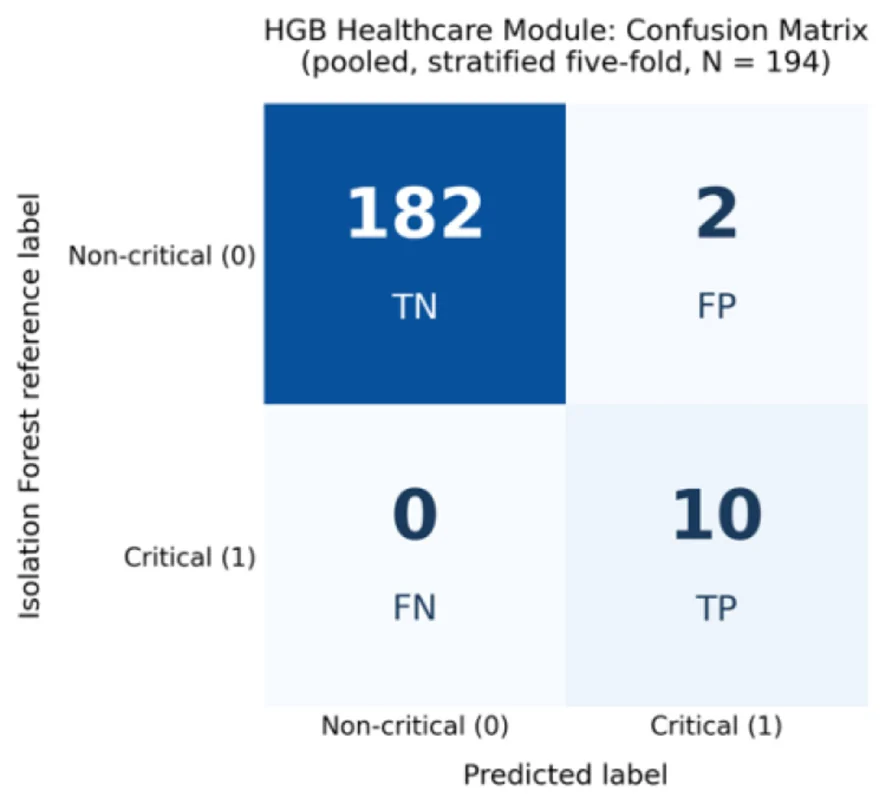
Confusion Matrix: Healthcare Module.

Figure 8 Stratified Five-Fold Cross Validation (*N* = 194) Confusion Matrix for Healthcare AI Module. The individual fold-level predictions were not saved and instead counts are reconstructed from the summary metrics at the per-fold level. This matrix is based on agreement with the labels generated by the Isolation Forest and not on clinically confirmed events of deterioration, and it does not solve the target-leakage problem mentioned previously in this section. The pooled counts (TN = 182, FP = 2, FN = 0, TP = 10) yield 98.97% accuracy, 83.33% precision, 100% recall, and 90.91% F1. The ratios in these pooled values are not identical to the fold-averaged means in Table 7 because it is not mathematically equivalent to averaging a ratio, and taking the ratio of the counts, particularly if there are only two positive cases in each fold. The exact 95% binomial interval for the recall is 0.6915 to 1.000 with ten detected positives out of ten. It is important to note that the observed detection rate of 1.000 should be interpreted as being consistent with perfect detection in a new population, rather than as evidence of such performance.

## 6 Discussion

The results support one main point. Running network security and anomaly-based patient-state screening in sequence on a single edge node meets both needs at once, without letting one weaken the other. It is worth explaining why each part of the design behaves the way it does. This distinguishes the present work from prior integrations of edge intelligence and network security, which typically execute both functions in parallel and reconcile their outputs afterward. The sequential ordering used here is a design decision that trades a small, bounded latency cost, quantified in Table 7, for a guarantee that a corrupted or adversarial input can never reach the clinical model undetected.

Table 9 situates the proposed framework against four recent Medical IoT intrusion detection systems. Each of the four achieves accuracy above 97%, comparable to or exceeding the security module reported in this work, which confirms that soft-voting over classical machine learning models remains competitive with more complex deep learning architectures such as transformers and CNN-LSTM hybrids on this class of problem. None of the four, however, report inference latency, and none integrate a clinical decision-making module alongside intrusion detection; each addresses network security in isolation. This supports the positioning stated in Section 1.2: the contribution of the present work is not a claim of superior detection accuracy over every recent method, but the sequential integration of network security and clinical inference within a single measured, latency-bounded pipeline, a combination not present in any of the compared systems.

**Table 9:** Comparison with recent Medical IoT security systems.

Study	Approach	Dataset	Reported Accuracy	Clinical module?	Reported latency	Practical limitation
FOID (Shaikh et al.) (44)	Recursive feature elimination + ML, SHAP explainability	ICU dataset; WUSTL-EHMS-2020	98.53%; 97.91%	No	Not reported	Security-only; no edge or clinical integration reported
MF-Transformer (Shaikh et al.) (45)	Memory-feedback LSTM fused into every Transformer layer	WUSTL-EHMS-2020; ECU-IoHT; X-IIoTID	99.88%/99.42%/99.12% (signature); 99.98%/99.71%/99.18% (anomaly)	No	Not reported	Transformer architecture; heavier inference cost than classical ML, not quantified in source
HCLR-IDS (Shaikh et al., DRL-based) (43)	CNN + LSTM + reinforcement learning (DQN, PPO)	CICIoMT2024	99.58% acc., 99.53% prec., 99.83% rec., 99.57% F1, AUC 0.99	No	Not reported	RL training and inference overhead typically substantial; not benchmarked for edge deployment
RCLNet (Shaikh et al.) (46)	RF feature selection + CNN-LSTM + attention	WUSTL-EHMS-2020	99.78%	No	Not reported	CNN-LSTM-attention stack; no reported edge latency or resource profile
Proposed (this work)	Soft-voting ensemble (Security) + HGB (Healthcare), sequential Zero-Trust gate	CICIDS-2017 (security); MIMIC-IV (clinical)	99.4% acc., 99.1% prec., 99.3% rec., 99.2% F1 (security); 97.8–98.97% (clinical, see Section 5)	Yes	HGB: 3.79 ms mean (Table 6)	Reported per-module accuracy is comparable to prior work; the proposed framework additionally reports directly measured inference latency, and is, to our knowledge, the only one of these systems that couples intrusion detection with clinical decision support in a single ordered pipeline

The security-first order is the core of the design. The Security AI Module screens every flow before the clinical model sees any data. This closes a weakness that affects most clinical edge systems, where a corrupted or adversarial stream can reach the model and change its output. In the proposed framework, such a stream is blocked at Stage 1 and never becomes a clinical decision. A parallel design cannot make this guarantee, because the clinical model would be reading data that has not yet been checked.

The selection of HGB also agrees with the data. The MIMIC-IV working set is sparse and non-representative, containing approximately 194 records, and approximately 5.15% positive cases. In such an environment, the HGB achieves a better regularization than the linear model does, and less overfitting than the internal binning of HGB. The most important clinical outcome is “recall of 1.000 in all folds”. If the monitor does not alarm for a normal reading, the damage resulting from an unalarmed critical case is much worse than from a false alarm, therefore complete recall is the most important property. This recall is being measured against recall labels generated by the Isolation Forest, not clinical events, so should be interpreted as a measure of the coherence of the labels, not diagnostic accuracy. From the security perspective, the soft voting ensemble has an advantage due to the fact that it's a mixture of models with different types of errors. Random Forest is already strong at 99.1% accuracy, and by allowing confidence models to dominate the rest, the ensemble is better than that. The gain is found in all accuracy, precision, recall and F1 score measures and is not limited to just one measure. The SMOTE step was required as if it weren't included, the common classes would have dominated the training data and prevented the rare classes from being learned. The latency results also point to a clear tuning option. The K-Nearest Neighbors member is the main cost in the security stage, since it scans the training set at prediction time. Removing or approximating it would cut the security latency sharply, with little loss of accuracy, because Random Forest already reaches 99.1% on its own.

Lastly, the additive latency model provides fair timing. Each stage is executed in sequence, as opposed to the maximum of 2 parallel paths. A benign flow has the full latency, and an attack ends early and is less costly. This puts the cost of the security gate on the table and finite, what a deployment engineer needs to believe the design.

## 7 Limitations and future work

### 7.1 Limitations

Consistent with the scope stated in Section 1, the governance and storage extension described in Section 7.2 is a conceptual design that has not been implemented or evaluated in this study. Eight limitations bound this study.

Firstly, the models were trained and profiled on a workstation and subsequently the edge stages were estimated for Raspberry Pi type hardware. Explicit power and thermal validation of a real ward with many sensors under continuous load.

Second, the evaluation used benchmark datasets, which were based on stable distributions. MIMIC-IV and CICIDS-2017 are retrospective data (not a real-time feed) and CICIDS-2017 is a labeled sample from a controlled testbed. Neither captures the drift, diversity, and attack behavior of a real hospital network. The actual numbers will vary, and the extent of this difference is only known once a prospective study is conducted.

Third, the clinical labels are based on an Isolation Forest, rather than a validated severity score. The module therefore does not carry out validated clinical diagnosis, but rather anomaly-based screening, and would require revalidation against outcomes labeled prospectively and confirmed by clinicians before use at the bedside.

Fourth, the network management stage is designed but not yet fully tuned. Its routing and QoS policy are described as part of the architecture, and its optimization is left to future work.

Fifth, this study does not experimentally measure the Healthcare AI module's sensitivity to corrupted or adversarial manipulated input. The threat model in Section 3.3 identifies this as a residual risk not covered by network-level Zero-Trust screening, but quantifying it is left to future work.

Sixth, unaware of this, the output of the early evaluation of the Healthcare AI module was left as the raw anomaly score of the Isolation Forest, a metric that was used as an input feature with the physiological statistics. The training label being a direct cutoff of this score caused target leakage. This was found in the revision process and fixed by omitting the anomaly score from the feature set; the revised results, without the leakage, are presented in Section 5. The original issue is disclosed here for transparency, as it had a material impact on an earlier version of the disclosed metrics.

Seventh, the original cross-validation split of the Healthcare AI module was done at the record level and not the patient level. Because a small number of patients in the MIMIC-IV working set contribute more than one record, a record-level split could place the same patient's data in both the training and test partitions of a fold. This was identified during revision and corrected using a patient-grouped stratified split, which guarantees that no patient's records are shared across the training and test sets; the corrected results are reported in Section 5.

Eighth, there are just 10 positive cases out of 194 records in the Healthcare AI module's evaluation set. Under the corrected, leakage-free and patient-grouped evaluation a reported recall of 1.000 is based on this small number. The 95% confidence interval for the recall is the range of 0.69 to 1.00, and the results should be interpreted as a sign of label coherence, but not as a guarantee of predictable clinical performance.

### 7.2 Future work

The governance and storage layer clearly is the next step that the framework was designed to transport. Each verified decision would be recorded on an audit-trail that would be impossible to tamper with on a permissioned Hyperledger Fabric ledger (21, 30). This is because bulk records, including long monitoring logs, will be stored off-chain on IPFS and only the content identifier and hash will be stored on the chain (24, 25). Retrieval would then check for integrity by comparing the hash on retrieval. Only clinicians with a role would be able to view a record (26) would be enforced by a smart contract. Once this layer is implemented, this layer will include the Δt_tx term in the latency model.

In addition, there are a number of possible implications of the constraints. A pilot test at a hospital would test the models under real traffic conditions and generate the labeled data required to revalidate the clinical module. From a detection level, the security module may be taken up to an active level for quarantining a compromised node or automatically updating firewall rules. Such an adaptive response is already possible with reinforcement-learning-based intrusion detectors for IoMT networks (43). Adversarial frameworks for other high-stakes tasks (e.g., computer vision) that are designed to be explainable can also be adapted to safeguard the Healthcare AI Module from adversarial input manipulation (43–47). No experiments have been conducted with the current evaluation to test the behavior of the Healthcare AI Module when faced with corrupted or adversarially manipulated vitals data, whether as a result of a network attack that bypasses the Security AI gate, or due to an adversarially manipulated sensor, which cannot be detected on principle by the Security AI gate. Future research will focus on a controlled perturbation experiment: the rate of clinical decision change with increasing random noise applied to the input features; the minimum required perturbation for the input features to be sufficiently noisy to misclassify a true critical as a non-critical decision, based on the model's feature importances. This would provide a direct measured indicator of the sensitivity of the Healthcare AI Module after corruption of the input, which is identified as a limitation but not yet measured in the present study. Federated learning, combined with lightweight encryption, would let several hospitals improve a shared model without exchanging patient records (36, 38, 40). Finally, a lighter consensus scheme, such as Proof-of-Authority, would reduce the ordering cost as the network grows across many hospitals (30, 35).

## 8 Conclusion

This article presented a dual-layer edge intelligence framework for secure, real-time Medical IoT. The framework runs two models in sequence on a single ward-level edge node. A Security AI Module, built on a soft-voting ensemble, screens every network flow first and acts as a Zero-Trust gate. Only verified traffic then reaches a Healthcare AI Module, built on Histogram Gradient Boosting, which classifies the patient's condition as critical or non-critical.

On CICIDS-2017, the security module reached 99.4% accuracy with a false-positive rate below 2%, ahead of five individual detectors. On MIMIC-IV, the clinical module reached a cross-validated accuracy of 98.97% and a ROC-AUC of 0.995, with complete recall across all folds, so no critical state was missed. Because the two stages run in sequence, the total edge latency is the sum of the stage times, and it stayed within the sub-second budget required for ICU monitoring.

The security-first order is the key idea. It ensures that no clinical decision is ever made on unverified data, which protects the clinical model from corrupted or adversarial input. A permissioned ledger with off-chain storage is set out as the next stage of the work, along with a prospective clinical pilot. Taken together, the results show that pairing intrusion detection with anomaly-based patient-state screening at the edge is a practical foundation for secure, real-time MIoT, pending prospective clinical validation before any diagnostic use.

## Data Availability

Publicly available datasets were analyzed in this study. MIMIC-IV is available through PhysioNet under a data-use agreement. CICIDS-2017 is publicly available from the Canadian Institute for Cybersecurity.

## References

[1] Ashfaq Z Rafay A Mumtaz R Zaidi SMH Saleem H Zaidi SAR . A review of enabling technologies for internet of medical things (IoMT) ecosystem. Ain Shams Eng J. (2022) 13:101660. doi: 10.1016/j.asej.2021.101660

[2] Ahmed SF Alam MSB Afrin S Rafa SJ Rafa N Gandomi AH. Insights into internet of medical things (IoMT): data fusion, security issues and potential solutions. Inf Fusion. (2024) 102:102060. doi: 10.1016/j.inffus.2023.102060

[3] Siqueira LP Batista CL Lui PH Kazienko JF Quincozes SE Quincozes VE. et al. A comprehensive survey on intrusion detection systems for healthcare 50. Sensors. (2025) 25:6261. doi: 10.3390/s25206261 41157315 PMC12567394

[4] Al-Otaibi S Ayouni S Sarwar N Irshad A Ullah F. AI-driven security framework for medical sensor networks: enhancing privacy and trust in smart healthcare systems. Cluster Comput. (2025) 28:408. doi: 10.1007/s10586-024-05049-3

[5] Nguyen DC Pathirana PN Ding M Seneviratne A. BEdgeHealth: a decentralized architecture for edge-based IoMT networks using blockchain. IEEE Internet Things J. (2021) 8:11743–57. doi: 10.1109/JIOT.2021.3058953

[6] Berguiga A Harchay A Massaoudi A. HIDS-IoMT: a deep learning-based intelligent intrusion detection system for the internet of medical things. IEEE Access. (2025) 13:32863–82. doi: 10.1109/ACCESS.2025.3543127

[7] Banaamah A Ahmad I. Intrusion detection in IoT using deep learning. Sensors. (2022) 22:8417. doi: 10.3390/s22218417 36366115 PMC9658941

[8] Dahou A Abd Elaziz M Chelloug SA Awadallah MA Al-Betar MA Al-Qaness MAA . Intrusion detection system for IoT based on deep learning and modified reptile search algorithm. Wirel Commun Mob Comput. (2022) 2022:6473507. doi: 10.1155/2022/6473507 37332528 PMC10275688

[9] Wang W Chen H Yang S Luo X Li D Wang J. A lightweight intrusion detection method for IoT based on deep learning and dynamic quantization. PeerJ Comput Sci. (2023) 9:*e*1569 doi: 10.7717/peerj-cs.1569 37810346 PMC10557502

[10] Finlayson SG Bowers JD Ito J Zittrain JL Beam AL Kohane IS. Adversarial attacks on medical machine learning. Science. (2019) 363:1287–9. doi: 10.1126/science.aaw4399 30898923 PMC7657648

[11] Ma X Niu Y Gu L Wang Y Zhao Y Bailey J ., Understanding adversarial attacks on deep learning based medical image analysis systems. Pattern Recognit. (2021) 110:107332 doi: 10.1016/j.patcog.2020.107332

[12] Thiele D Rodseth R Friedland R Berger F Mathew C Maslo C . Machine learning models for the early real-time prediction of deterioration in intensive care units—a novel approach to the early identification of high-risk patients. J Clin Med. (2025) 14:350. doi: 10.3390/jcm14020350 39860355 PMC11766095

[13] Alhashmi SM. Knowledge management, health data, and advanced data analytics to expedite solutions in the healthcare industry. In: Digital Healthcare, Digital Transformation and Citizen Empowerment in Asia-Pacific and Europe for a Healthier Society. London: Academic Press (2025) 231–47. doi: 10.1016/B978-0-443-30168-1.00005-0

[14] Amintoosi H Nikooghadam M Shojafar M Kumari S Alazab M. Slight: A lightweight authentication scheme for smart healthcare services. Comput Electr Eng. (2022) 99:107803. doi: 10.1016/j.compeleceng.2022.107803

[15] Ahmed AA Abdullah TA. An enhanced XGBoost-based framework for efficient multi-class cyber threat detection in industrial IoT networks. Technologies. (2026) 14:274. doi: 10.3390/technologies14050274

[16] Grajales-Bustamante JD Murillo-Escobar J Delgado-Trejos E. Multilevel intrusion detection system based on machine learning techniques. J Eng. (2026) 2026:7142912. doi: 10.1155/je/7142912

[17] Asante IO Wu L. Enhancing cybersecurity through hybrid blockchain-enabled intrusion detection systems: a machine learning approach. Peer-to-Peer Netw Appl. (2025) 18:268. doi: 10.1007/s12083-025-01907-y

[18] Pinto D Amorim I Maia E Praça I. A review on intrusion detection datasets: tools, processes, and features. Comput Netw. (2025) 262:111177. doi: 10.1016/j.comnet.2025.111177

[19] Maftei AA Petrariu AI Popa V Lavric A. A blockchain framework for scalable, high-density IoT networks of the future. Sensors. (2025) 25:2886. doi: 10.3390/s25092886 40363323 PMC12074401

[20] Villegas-Ch W Gutierrez R Maldonado Navarro A Mera-Navarrete A. Lightweight blockchain for authentication and authorization in resource-constrained IoT networks. IEEE Access. (2025) 13:48047–67. doi: 10.1109/ACCESS.2025.3551261

[21] Ouadrassi NL Boukhryss MS Azmani A Hassani KJ. Configuration driven performance analysis of Hyperledger Fabric: an empirical study. IEEE Access. (2026) 14:48145–56. doi: 10.1109/ACCESS.2026.3677633

[22] Pongnumkul S Siripanpornchana C Thajchayapong S. Performance analysis of private blockchain platforms in varying workloads, In: Proc. 26th Int. Conf. Computer Communication and Networks (ICCCN) (2017), p. 1–6. doi: 10.1109/ICCCN.2017.8038517

[23] Dinh TTA Wang J Chen G Liu R Ooi BC Tan KL. BLOCKBENCH: A framework for analyzing private blockchains. In: Proc. ACM Int. Conf. Management of Data (SIGMOD) (2017), p. 1085–1100 doi: 10.1145/3035918.3064033

[24] Benet J. IPFS—Content addressed, versioned, P2P file system. arXiv [preprint] (2014)

[25] Mishra RK Yadav RK. Nath P.Integration of blockchain and IPFS: Healthcare data management and sharing for IoT environment. Multimed Tools Appl. (2025) 84:27229–50. doi: 10.1007/s11042-024-20092-3

[26] Upadhyay V Kokila J Vaish A. A dual framework for optimized data storage and retrieval using lightweight Python blockchain and scalable smart contracts with IPFS. Peer-to-Peer Netw Appl. (2026) 19:49. doi: 10.1007/s12083-025-02192-5

[27] Bezanjani BR Ghafouri SH Gholamrezaei R. Privacy preserving healthcare data in IoT: A synergistic approach with deep learning and blockchain. J Supercomput. (2025) 81:533. doi: 10.1007/s11227-025-06980-x

[28] Aboshosha BW Zayed MM Khalifa HS Ramadan RA. Enhancing internet of things security in healthcare using a blockchain driven lightweight hashing system. Beni Suef Univ J Basic Appl Sci. (2025) 14:56. doi: 10.1186/s43088-025-00644-8

[29] Wazid M Gope P BACKM-EHA. A novel blockchain-enabled security solution for IoMT-based e-healthcare applications. ACM Trans Internet Technol. (2023) 23:1–28. doi: 10.1145/3511898

[30] Kokate S Shrawankar U. IoT data transmission security using blockchain with a trust-weighted proof of authority consensus mechanism in healthcare. Trans Emerg Telecommun Technol. (2025) 36: *e*70139 doi: 10.1002/ett.70139

[31] Chawla NV Bowyer KW Hall LO Kegelmeyer WP. SMOTE: synthetic minority over-sampling technique. J Artif Intell Res. (2002) 16:321–57, doi: 10.1613/jair.953

[32] Johnson AEW Bulgarelli L Shen L Gayles A Shammout A Horng S . MIMIC-IV, a freely accessible electronic health record dataset. Sci Data. (2023) 10:1. doi: 10.1038/s41597-022-01899-x 36596836 PMC9810617

[33] Zhao J Xue K Li M Xue Y Huang Y IFAD. Privacy-preserving isolation forest-based anomaly detection in public cloud environments. IEEE Trans Inf Forensics Secur. (2026) 21:3828–41. doi: 10.1109/TIFS.2026.3678112

[34] Hastie T Tibshirani R Friedman J. Random forests. In: The Elements of Statistical Learning: Data Mining, Inference, and Prediction, 2nd Edn. New York, NY: Springer (2009). p. 587–604 doi: 10.1007/978-0-387-84858-7_15

[35] Ongaro D Ousterhout J. In search of an understandable consensus algorithm. In: Proc. USENIX Annual Technical Conference (USENIX ATC) (2014). p. 305–319.

[36] Elayan H Aloqaily M Guizani M Hamdi M. Blockchain-enabled federated learning for secure internet of medical things: recent advances, challenges, and future directions. IEEE Internet Things J. (2024). doi: 10.1109/JIOT.2023.3263598

[37] Qammar A Karim A Ning H Ding J. Securing federated learning with blockchain: a systematic literature review. Artif Intell Rev. (2023) 56:3951–85. doi: 10.1007/s10462-022-10271-9 36160367 PMC9483378

[38] Rieke N Hancox J Li W Milletarì F Roth HR Albarqouni S . The future of digital health with federated learning. NPJ Digit Med. (2020) 3:119. doi: 10.1038/s41746-020-00323-1 33015372 PMC7490367

[39] Alzubi JA Alzubi OA Singh A Ramachandran M. Cloud-IIoT-based electronic health record privacy-preserving by CNN and blockchain-enabled federated learning. IEEE Trans Ind Inform. (2023) 19:1080–7. doi: 10.1109/TII.2022.3189170

[40] Ramani R Rosline Mary A Edwin Raja S Arun Shunmugam D. Optimized data management and secured federated learning in the internet of medical things (IoMT) with blockchain technology. Biomed Signal Process Control. (2024) 93:106213. doi: 10.1016/j.bspc.2024.106213

[41] Rose S Borchert O Mitchell S Connelly S. Zero Trust Architecture. In: National Institute of Standards and Technology, Gaithersburg, MD, USA, NIST Special Publication 800-207 (2020) doi: 10.6028/NIST.SP.800-207

[42] Srivastava V Motani M. Cross-layer design: a survey and the road ahead. IEEE Commun Mag. (2005) 43:112–9. doi: 10.1109/MCOM.2005.1561928

[43] Shaikh JA Wang C Sima MWU Arshad M Owais M Hassan DSM . A deep reinforcement learning-based robust intrusion detection system for securing IoMT healthcare networks. Front Med. (2025) 12:1524286. doi: 10.3389/fmed.2025.1524286 40309737 PMC12041770

[44] Shaikh JA Wang C Owais M Zia U Sima MWU Arshad M. FOID: A feature-optimized intrusion detection system for securing IoMT healthcare networks. In: Proc. 18th Int. Conf. Open Source Systems and Technologies (ICOSST) (2024). p. 1–7. doi: 10.1109/ICOSST64562.2024.10871156

[45] Shaikh JA Wang C Sima MWU Arshad M Rathore WUA. Memory feedback transformer based intrusion detection system for IoMT healthcare networks. Internet Things. (2025) 32:101597. doi: 10.1016/j.iot.2025.101597

[46] Shaikh JA Wang C Muhammad WUS Arshad M Owais M Alnashwan RO . Muthanna, RCLNet: An effective anomaly-based intrusion detection for securing the IoMT system. Frontiers in Digital Health. (2024) 6:1467241. doi: 10.3389/fdgth.2024.1467241 39421756 PMC11484052

[47] Shaikh JA Aldrees A Eshmawi AA Umer M Cascone C Huo Y. SentriShield-VQ: an explainable variational quantum adversarial defense framework with interpretable attribution for secure computer vision under multi-vector attacks. Image Vis Comput. (2026) 174:106140. doi: 10.1016/j.imavis.2026.106140

